# Effects and Mechanisms of Dietary Natural Products on Ischemic Stroke: An Updated Review

**DOI:** 10.1002/fsn3.71324

**Published:** 2025-12-10

**Authors:** Kai‐Yi Zhong, Yong Zhang, Nai‐Dong Wang, Guang‐Wen Li, Xian‐Jun Zhang, Zhi‐Jun Yang

**Affiliations:** ^1^ Department of Neurology The Affiliated Hospital of Qingdao University Qingdao China; ^2^ Institute of Fruit Tree Research, Guangdong Academy of Agricultural Sciences, Key Laboratory of South Subtropical Fruit Biology and Genetic Resource Utilization, Ministry of Agriculture and Rural Affairs, Guangdong Provincial Key Laboratory of Science and Technology Research on Fruit Tree Guangzhou China

**Keywords:** dietary natural products, gut microbiota, inflammation, ischemic stroke, molecular mechanisms, neuroprotection

## Abstract

Ischemic stroke ranks among the primary contributors to mortality and prolonged disability globally, representing a significant public health challenge. Some clinical drugs for the treatment of ischemic stroke have significant side effects. Therefore, exploring effective therapeutic strategies is crucial. Some dietary natural products, such as fruits, vegetables, teas, herbs, nuts, probiotics and prebiotics, exert potential neuroprotective effects on ischemic stroke. The underlying mechanisms of action include suppressing oxidative stress, inhibiting inflammation, alleviating excitotoxicity, promoting angiogenesis, protecting blood–brain barrier, regulating gut microbiota, attenuating apoptosis, inhibiting autophagy, suppressing platelet aggregation and thrombosis, and improving mitochondrial function. This review mainly summarizes recent advancements in the potential therapeutic effects and mechanisms of dietary natural products on ischemic stroke. Additionally, it highlights future research directions, including the synergistic effects of combining dietary natural products, as well as the incorporation of nanotechnology to enhance bioavailability and targeted delivery. Overall, this review provides a useful reference for the application of dietary natural products in the prevention and management of ischemic stroke.

## Graphical abstract

This review mainly summarizes the effects and mechanisms of dietary natural products on ischemic stroke, and highlights future research directions. Overall, this review provides a useful reference for the application of dietary natural products in the prevention and management of ischemic stroke.

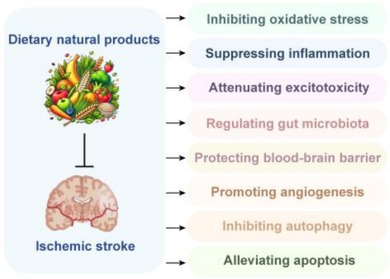

## 1 Introduction

Stroke ranks as the second most common cause of death globally (Hilkens et al. 2024). Stroke consists of two parts: ischemic stroke, which accounts for approximately 87% of all strokes, and hemorrhagic stroke (Benjamin et al. 2017). Ischemic stroke occurs due to abnormal blood flow to the brain, which triggers a series of events caused by oxygen and nutrient deficiencies, resulting in neuronal damage and death, leading to hemiplegia, paraplegia, dysarthria, and mild paralysis. The key molecular mechanisms include excitotoxicity, acidosis, neuroinflammation, oxidative stress, disruption of the blood–brain barrier, mitochondrial dysfunction, apoptosis, and autophagy (Kakarla et al. 2024; Majumder 2024; Salaudeen et al. 2024). With its high rates of incidence, morbidity, and mortality, ischemic stroke imposes a substantial burden on both society and the economy, thus making it a significant public health concern (Wang, Fang, et al. 2021). Grasping the underlying mechanisms of ischemic stroke is essential for the creation of innovative management strategies. Current clinical treatments for ischemic stroke include intravenous thrombolytic therapy and interventional thrombectomy (Hankey 2017; Kleindorfer et al. 2021). However, these treatments have a narrow therapeutic window and carry a high risk of hemorrhagic complications (Frank et al. 2022; Wang, Fang, et al. 2021). Some drugs, such as edaravone, are used clinically to treat ischemic stroke, but they have significant side effects, such as acute renal failure (Lapchak 2010). Therefore, there is a growing research hotspot in exploring dietary natural products for the prevention and management of ischemic stroke.

Recent literatures have shown that some dietary natural products have preventive and therapeutic effects on some diseases, such as Alzheimer's disease (Yang et al. 2023), cardiovascular diseases (Jin Cheng et al. 2023; Zhou et al. 2022), obesity (Shang et al. 2021; Xu et al. 2023), diabetes (Cheng et al. 2024; Yang et al. 2025), cancer (Wu et al. 2023; Yang, Huang, et al. 2022), depression and anxiety (Wu, Li, et al. 2022; Yang et al. 2025). Furthermore, numerous studies have indicated that dietary natural products have certain benefits for ischemic stroke (Liu, Wu, et al. 2024; Shaheryar et al. 2023; Tian et al. 2020; Yang, Du, et al. 2022; Yuan et al. 2021). A meta‐analysis showed that higher total dietary fiber was related to a lower incidence of ischemic stroke (hazard ratio (HR), 0.83; 95% confidence interval (95% CI), 0.79–0.88) (Li, Hao, and Hu 2023). In addition, blueberry extract could improve ischemic stroke by maintaining the gut barrier and regulating gut microbiota via enriching *Prevotella*, *Prevotellaceae* and *Blautia* (Wang et al. 2024). Moreover, betaine could protect the brain from ischemia–reperfusion (IR) injury in SD rats through inhibiting oxidative stress, suppressing inflammation and mitigating neuronal apoptosis (Li, Qu, et al. 2022).

In this review, we collected literature published in the past 5 years, summarized the effects of dietary natural products on ischemic stroke relying on the results of epidemiological, experimental and clinical research, and discussed their potential mechanisms of action. Additionally, we also discussed the future research directions on dietary natural products for the prevention and management of ischemic stroke. This review aims to promote further research and the application of dietary natural products in the prevention and management of ischemic stroke.

## 2 Epidemiological Studies

Some epidemiological literature has found that several dietary natural products are associated with ischemic stroke risk (Table 1). For instance, a cohort‐based prospective research found that every 100 g increase in daily intake of coarse grains was associated with a 13% decrease in the risk of ischemic stroke (HR, 0.87; 95% CI, 0.81–0.94) (Yang, Du, et al. 2022). Furthermore, a cohort study of 57,053 participants revealed that replacing potatoes with fruiting vegetables could reduce the risk of ischemic stroke (HR, 0.86; 95% CI, 0.76–0.97) (Hansen et al. 2021). Moreover, a prospective cohort study of 179,827 veterans found that consuming ≥ 5 times/week of nuts was correlated with a decreased risk of ischemic stroke (HR, 0.81; 95% CI, 0.71–0.92) (Ivey et al. 2021). Another prospective cohort study of 74,793 participants revealed that peanut consumption was correlated with a reduced risk of ischemic stroke (HR, 0.80; 95% CI, 0.71–0.90) (Ikehara et al. 2021). Besides, a prospective cohort study revealed that compared with those who never consumed tea, people with higher consumption of tea (occasionally (HR, 0.96; 95% CI, 0.93–0.98), weekly (HR, 0.94; 95% CI, 0.90–0.995) and daily (HR, 0.92; 95% CI, 0.89–0.96)) had a lower risk of ischemic stroke (Tian et al. 2020). Moreover, a prospective cohort study of 365,682 participants found that compared with those who never drank tea and coffee, those who drank 2–3 cups of coffee and 2–3 cups of tea per day had a decreased risk of ischemic stroke (HR, 0.62; 95% CI, 0.51–0.75) (Zhang et al. 2021). However, a case–control study of 13,462 individuals diagnosed with ischemic stroke and 13,488 healthy controls indicated that no significant association was found between coffee consumption and ischemic stroke (OR, 1.32; 95% CI, 1.00–1.74). Conversely, tea drinking was shown to be inversely related to the risk of ischemic stroke (OR, 0.81; 95% CI, 0.68–0.98) (Smyth et al. 2024).

**TABLE 1:** Effects of dietary natural products on ischemic stroke from epidemiological studies.

Name	Study type	Participants	Effects	Ref.
Grains				
Coarse grains	Prospective cohort study	> 0.5 million adults	Each 100 g/day increase in intake of coarse grains was associated with 13% lower risks (HR, 0.87; 95% CI, 0.81–0.94)	Yang, Du, et al. (2022)
Fruits and vegetables				
Fruiting vegetables	Cohort study	57,053 participants	Replacing potatoes with fruiting vegetables could reduce the risk of ischemic stroke (HR, 0.86; 95% CI, 0.76–0.97)	Hansen et al. (2021)
Nuts				
Nut	Prospective cohort study	179,827 veterans	Associated with a decreased risk of ischemic stroke (Consumption nuts ≥ 5 times per week vs. never consuming nuts: HR, 0.81; 95% CI, 0.71–0.92); Consumption of peanut butter was not associated with the risk of ischemic stroke	Ivey et al. (2021)
Peanut	Prospective cohort study	74,793 participants	Associated with lower risk of ischemic stroke (the highest vs. lowest quartiles of peanut consumption: HR, 0.80; 95% CI, 0.71–0.90, *p*‐trend = 0.002)	Ikehara et al. (2021)
Teas				
Tea	Prospective cohort study	487,377 participants	Higher consumption of tea is associated with a reduced risk of ischemic stroke (Consumed tea occasionally, weekly and daily vs. never consumed tea: HR, 0.96; 95% CI, 0.93–0.98, HR, 0.94; 95% CI, 0.90–0.995; HR, 0.92; 95% CI, 0.89–0.96)	Tian et al. (2020)
Coffee and teas				
Coffee and tea	Prospective cohort study	365,682 participants	Associated with a lower risk of ischemic stroke (drinking 2–3 cups of coffee and 2–3 cups of tea per day vs. not drinking tea and coffee: HR, 0.62; 95% CI, 0.51–0.75, *p* < 0.001)	Zhang et al. (2021)
Coffee and tea	Case–control study	13,462 cases and 13,488 controls	No association between coffee intake and ischemic stroke (OR, 1.32; 95% CI, 1.00–1.74); Tea consumption was associated with a decreased risk of ischemic stroke (OR, 0.81; 95% CI, 0.68–0.98)	Smyth et al. (2024)

Abbreviations: 95% CI, 95% confidence interval; HR, hazard ratio; OR, odds ratio.

In short, some dietary natural products, such as coarse grains, fruiting vegetables, teas, nuts and peanuts, could reduce the risk of ischemic stroke. In the future, more high‐quality and large‐sample‐size epidemiological studies should be carried out to verify the association between more dietary natural products and ischemic stroke.

## 3 Experimental Studies

**FIGURE 1:**
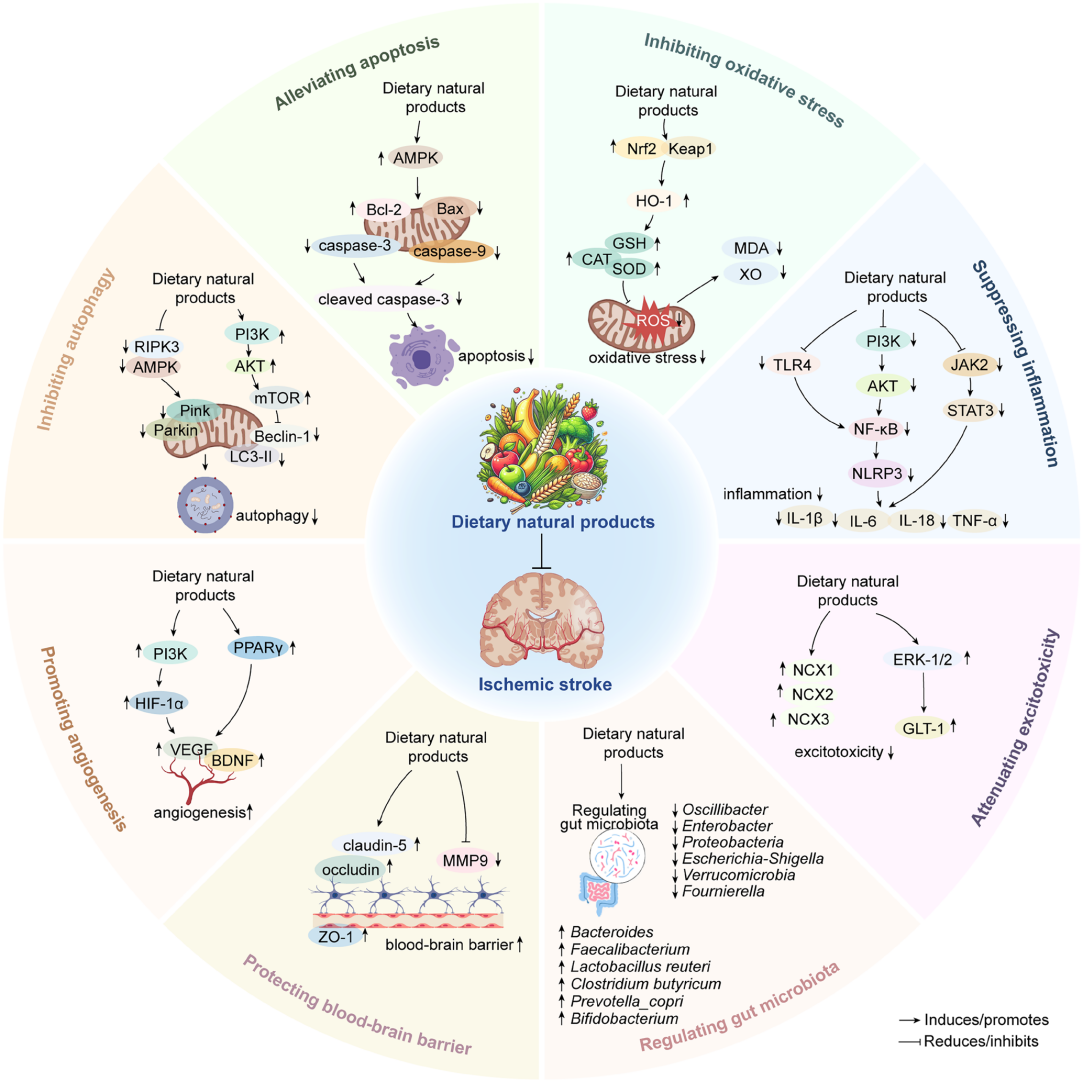
The effects and mechanisms of dietary natural products on ischemic stroke. It mainly includes inhibiting oxidative stress, suppressing inflammation, attenuating excitotoxicity, regulating gut microbiota, protecting blood–brain barrier, promoting angiogenesis, inhibiting autophagy and alleviating apoptosis. ↑ represents increase. ↓ represents decrease. AKT, protein kinase B; AMPK, AMP‐activated protein kinase; Bax, Bcl‐2 associated X protein; Bcl‐2, B‐cell lymphoma‐2; BDNF, brain‐derived neurotrophic factor; Beclin‐1, myosin‐like Bcl‐2 interacting protein; caspase‐3, cysteinyl aspartate specific proteinase 3; caspase‐9, cysteinyl aspartate specific proteinase 9; CAT, catalase; ERK, extracellular signal‐regulated protein kinase; GLT‐1, glutamate transporter 1; GSH, glutathione; HIF‐1α, hypoxia inducible factor 1 subunit alpha; HO‐1, heme oxygenase‐1; IL‐1β, interleukin‐1β; IL‐6, interleukin‐6; IL‐18, interleukin‐18; JAK2, janus kinase 2; Keap1, kelch‐like epichlorohydrin‐associated protein 1; LC3‐II, microtubule‐associated protein light chain 3 II; MDA, malondialdehyde; MMP9, matrix metallopeptidase 9; mTOR, mechanistic target of rapamycin kinase; NCX, Na^+^/Ca^2+^ exchanger; NLRP3, NLR family pyrin domain containing 3; NF‐κB, nuclear factor kappa‐B; Nrf2, nuclear factor‐erythroid 2‐related factor‐2; PI3K, phosphoinositide 3 kinase; PPARγ, peroxisome proliferator‐activated receptor gamma; RIPK3, receptor interacting serine/threonine kinase 3; ROS, reactive oxygen species; SOD, superoxide dismutase; STAT3, signal transducer and activator of transcription 3; TLR4, toll‐like receptor 4; TNF‐α, tumor necrosis factor α; VEGF, vascular endothelial growth factor; XO, xanthine oxidase; ZO‐1, zonula occludens‐1.

Accumulating evidence suggests that some dietary natural products have different effects and mechanisms on ischemic stroke, which will be discussed in detail below (Table 2 and Figure 1).

**TABLE 2:** Effects and mechanisms of dietary natural products on ischemic stroke from experimental studies.

Name	Components	Study type	Model	Dose and method	Main effects and related mechanisms	Ref.
Vegetables						
*Smyrnium olusatrum* L.	Isofuranodiene	In vivo	Brachiocephalic artery occlusion model in Wistar rats	Intraperitoneal injection: 40 mg/kg at 30 min before surgery	Shortened the behavioral recovery period, decreased the levels of MDA, TNF‐α and IL‐1β, and the expression of p‐NF‐κB/NF‐κB	Yousefi‐Manesh et al. (2021)
Celery	Apigenin	In vivo	pMCAO model in SD rats	Gavage: 30, 60 and 120 mg/kg/day for 7 days before surgery	Improved the volume of cerebral infarction, reduced parthanatos, ameliorated neurological deficits, reduced brain edema, decreased apoptosis, by inhibiting oxidative stress, suppressing PARP1/AIF pathway, and reducing DNA damage	Ping et al. (2024)
Cruciferous vegetables	Indole‐3‐carbinol	In vivo and in vitro	MCAO/R model in SD rats; OGD/R model in HAPI cells	Intraperitoneal injection: 150 mg/kg/day for 3 days after surgery; Culture: pretreated with 50 μM/mL for 4 h before OGD/R	Promoted neurological recovery, reduced inflammation, decreased neuronal loss, inhibited apoptosis via inhibiting the expression of caspase‐3, caspase‐9 and Bax, and increasing the expression of Bcl‐2	Peng et al. (2024)
Fruits						
Grape	Narirutin‐rich fraction from grape fruit peel	In vivo	BCCAO model in Wistar rats	Oral administration: 150 and 300 mg/kg/day for 7 days before surgery	Improved neurobehavioral alterations, reduced MDA level, rose the activities of CAT and SOD	Patel et al. (2022)
*Euterpe oleracea* Mart. fruits	Lyophilized extract of fruits	In vivo	MCAO model in Wistar rats	Gavage: 200 mg/kg/day for 14 days, at 4.5 h after surgery	Improved the behavioral outcome, grew the number of surviving neurons, and decreased the size of the area of infarction	Teixeira et al. (2023)
Olive	Olive oil and olive leaf extract	In vivo	MCAO model in Wistar rats	Gavage (olive oil): 0.25, 0.50 and 0.75 mL/kg for 30 days before surgery; Oral administration (leaf extract): 50, 75 and 100 mg/kg/day for 30 days before surgery	Reduced the neurological and infarction score, increased NCXs (NCX1, NCX2 and NCX3) expression	Khaksar et al. (2025)
Blueberry extract	Rutin, procyanidin B1, quercetin, chlorogenic acid, D‐catechin, L‐epicatechin, and cyanidin‐3‐O‐glucoside	In vivo	MCAO model in SD rats	Gavage: 100 and 400 mg/kg/day for 14 days after surgery	Reduced neurological deficits and infarct volume, improved neurological function, gut‐brain barrier and gut microbiota, increased the abundance of *Prevotella*, *Prevotellaceae* and *Blautia*	Wang et al. (2024)
Teas						
*Lithocarpus Polystachyus* Rehd.	/	In vivo	MCAO model in SD rats	Gavage: 200, 400 and 600 mg/kg/day for 7 days before surgery	Alleviated neuronal loss, reduced oxidative stress, inhibited neuroinflammation by suppressing PI3K/AKT/NF‐κB pathway and modulating NLRP3‐mediated pyroptosis	Liu, Wu, et al. (2024)
Green tea	EGCG	In vivo and in vitro	MCAO/R model in C57BL/6 mice; OGD/R model in HT22 cells	Injected slowly (0.1 μL/min) into the right ventricle: 1 μg/μL, 4 μL at 1 h after MCAO/R; Culture: 20 μM for 2 and 4 h during reoxygenation	Protected poststroke neuronal loss, and reduced the infarct volume via inhibiting autophagy by the AKT/AMPK/mTOR pathway	Wang et al. (2022)
Herbs						
*Withania somnifera*	Withanolide A	In vivo	BCCAO model in Swiss albino mice	Intranasal administration: 1, 5 and 10 mg/kg after reperfusion	Decreased number of necrotic and apoptotic cells, and restored BBB disruption and cerebral oedema	Mukherjee et al. (2020)
*Rheum palmatum* L.	Emodin	In vivo and in vitro	MCAO model in SD rats; OGD/hypoxia model in PC12 cells	Intraperitoneal injection: 15 mg/kg; Culture: 10 μM for 4 and 24 h	Induced the expression of GLT‐1 and Bcl‐2, inhibited ROS generation, reduced glutamate toxicity, and suppressed neuronal apoptosis by activating ERK‐1/2 signaling pathway	Leung et al. (2020)
*Anchusa Italica*	Hydroalcoholic Extract	In vivo	Global cerebral artery occlusion model in Wistar rats	Intraperitoneal injection: 50 and 100 mg/kg for 10 days, 24 h after surgery	Increased the mRNA expression of *BDNF*, decreased the mRNA expression of *iNOS*, and increased the levels of MDA and NO	Asgharzade et al. (2020)
*Garcinia indica* fruit rind	Garcinol	In vivo and in vitro	MCAO/R model in SD rats; OGD/R model in PC12 cells	Treatment: 5, 10 and 20 mg/kg/day for 3 days; Culture: 2.5, 5 and 10 μM for 24 h before OGD/R	Decreased neurological deficit score and a smaller infarct size, suppressed the production of IL‐1β, TNF‐α and IL‐6, rose SOD activity, and reduced the levels of NO and MDA by suppressing TLR4/NF‐κB signal pathway	Kang et al. (2020)
*Tribulus terrestris* L. fruit	Gross saponins	In vivo and in silico	MCAO model in SD rats	Tail vein injection: 3 mg/kg/day for 3 days, before surgery, 24 h after MCAO surgery	Regulated metabolic pathways, and bound to the HSD11B1 and AR	Guo et al. (2020)
*Tribulus terrestris* L. fruit	Gross saponins	In vivo	MCAO model in SD rats	Tail vein injection: 3 mg/kg/day for 3 days, before surgery, 24 h after MCAO surgery	Regulated the complement and coagulation cascade	Wang, Guo, et al. (2021)
*Tribulus terrestris* L. fruit	Fruit extract	In vivo	MCAO model in Wistar rats	Tail vein injection: 3 mg/kg/day for 3 days, before surgery, 24 h after MCAO surgery	Elevated platelet cytosolic Ca^2+^, and activated platelet	Xu et al. (2022)
*Salvia miltiorrhiza* Bunge	Salvianolic acid B	In vivo	MCAO model in C57BL/6 mice	Oral administration: 5, 15 and 45 mg/kg at 3 and 6 h after commencing MCAO	Decreased infarct volumes and edema indices, reduced the levels of TNF‐α and IL‐1β, inhibited the protein expression of MnSOD, and reduced ROS	Lim et al. (2020)
*Salvia miltiorrhiza* Bunge	Salvianolic acid C	In vivo and in silico	MCAO model in SD rats	Intraperitoneal injection: 20 mg/kg/day for 7 days after MCAO surgery	Inhibited inflammation and promoted angiogenesis via elevating the expression of PPARγ, and suppressing the expression of VEGFR2, MMP1 and IGF1	Yang, He, et al. (2021)
*Salvia miltiorrhiza* Bunge	Phenolic acids	In vivo and in silico	Autologous thrombus stroke model in SD rats	Intragastrical administration: 10 mg/kg for 3 days before surgery, twice a day in the morning and evening	Decreased neurobehavioral scores, inhibited platelet aggregation, protected tight junction proteins, and reduced infarct size	Liu et al. (2023)
*Astragalus mongholicus* Bunge	Astragaloside IV	In vivo	tMCAO model in SD rats	Intraperitoneal injection: 40 mg/kg/day for 14 days after MCAO	Enhanced neurogenesis, angiogenesis and neurological functional recovery, promoted the transformation of microglia/macrophage from M1 to M2 phenotype, and increased expression of PPARγ, BDNF, IGF1 and VEGF	Li, Gan, et al. (2021)
*Tetrapleura tetraptera*	Fruit extract	In vivo	BCCAO/R model in Wistar rats	Oral administration: 50, 100 and 200 mg/kg/day for 7 days before surgery	Reduced behavioral deficits, enhanced GSH level, increased glutamine synthetase, CAT and SOD activities, reduced XO activity and TBARS level, decreased LDH and MPO activity, and attenuated disturbances in Na^+^ and K^+^ levels	Saliu et al. (2021)
*Trillium tschonoskii* Maxim. rhizome	Total saponins	In vivo	MCAO model in SD rats	Intragastrical administration: 33, 65 and 130 mg/kg/day for 15 days, at post‐stroke 6 h	Rose post‐stroke functional recovery, improved axonal regeneration, and amplified endogenous oligodendrogenesis by modulating GSK‐3/β‐catenin/CRMP‐2 pathway	Yang, Li, et al. (2021)
*Thymus quinquecostatus* Celak.	Polyphenol‐rich fraction extract	In vivo	tMCAO model in SD rats	Intragastrical administration: 70, 140 and 210 mg/kg/day for 7 days before operation	Ameliorated neurological deficit, improved the cellular morphology, modulated oxidative stress, reduced infarction rate, and suppressed the apoptosis of the neurons through activating the Keap1/Nrf2/HO‐1 signaling pathway	Fan et al. (2021)
Rabdosia rubescens	Oridonin	In vivo and in vitro	tMCAO model in C57BL/6N mice; OGD/R model in bEND.3 cells	Intraperitoneal injection: 20 mg/kg/day for 3 days after reperfusion; Culture: 1, 3 and 10 μM during reperfusion	Repaired BBB integrity, elevated tight junction proteins expression, reduced infarct volume, inhibited neuroinflammation, suppressed the infiltration of periphery inflammatory cells, and alleviated oxidative stress through promoting the AKT/GSK3β/Fyn/Nrf2 pathway	Li, Cheng, et al. (2021)
Rabdosia rubescens	Oridonin	In vivo and in vitro	tMCAO model in C57BL/6 mice; OGD/R model in N2a cells	Intraperitoneal injection: 5, 10 and 20 mg/kg during reperfusion; Culture: 1, 3 and 6 μM during reoxygenation	Attenuated neuronal loss and apoptosis, and alleviated excessive mitophagy via reducing the phosphorylation of AMPK by inhibiting RIPK3	Li, Song, et al. (2023)
Rhizoma Coptidis	Berberine	In vitro	OGD model in rat hippocampal cells	Culture: 0.5 μg/mL for 6 h before OGD	Inhibited pyroptosis via up‐regulating PPARγ to restrain NF‐κB	Zhao et al. (2021)
*Pueraria* lobate Radix	Daidzein	In vivo	MCAO model in ICR rats	Intraperitoneal injection: 10, 20 and 30 mg/kg/day for 2 weeks before surgery	Enhanced neurological deficits, elevated striatal dopamine and its metabolite levels, and improved infarct volume and brain edema through regulating the AKT/mTOR/BDNF channel	Zheng et al. (2021)
Red clover	Formononetin	In vivo	MCAO model in SD rats	Administration: 30 mg/kg/day for 3 days after surgery	Alleviated the neurological deficit, decreased the volume of cerebral infarction, reduced inflammatory factors, and ameliorated the pathological state of brain tissues through inhibiting JAK2/STAT3 pathway	Yu et al. (2022)
*Viola odorata*	Hydroalcoholic extract	In vivo	MCAO model in Wistar rats	Intragastrical administration: 25, 50 and 75 mg/kg/day for 30 days before surgery	Reduced the infarct volumes through inhibiting the expression of *NF‐κB* and *VCAM‐1*	Karimifar et al. (2022)
*Lithospermum erythrorhizon*	Monomethyl lithospermate	In vivo and in vitro	MCAO model in SD rats; OGD/R model in SHSY‐5Y cells	Intragastrical administration: 72.4 μM/kg/day for 14 days before surgery; Culture: 5, 10 and 20 μM for 12 h during reoxygenation	Improved the neurological damage, suppressed apoptosis, improved neurological deficit score and hippocampal neuron damage, and reduced oxidative stress via activating the PI3K/AKT pathway	Yang, Chen, et al. (2022)
*Verbena offcinalis* L.	Cornin	In vivo and in vitro	MCAO model in SD rats; OGD/R model in U87 cells	Tail vein injection: 2.5, 5 and 10 mg/kg after surgery for 15 min; Culture: 10, 30 and 100 nM before hypoxia for 48 h	Reduced the cerebral infarction volume, improved neurological recovery, inhibited BBB leakage, inhibited apoptosis and autophagy, and reduced the expression of Beclin‐1 and LC3‐II via stimulating the PI3K/AKT/mTOR pathway	Lan et al. (2022)
*Polygonum multiflorum* Thunb.	Tetrahydroxy stilbene glucoside	In vivo	BCCAO/R model in C57BL/6 mice	Subcutaneous injection: 100 mg/kg at 2 and 12 h before surgery, 50 mg/kg at 0, 3 and 6 h and 100 mg/kg at 22, 48 and 70 h after surgery	Eliminated brain injuries through promoting M2 macrophage polarization by regulating conformation‐dependent intracellular distribution of PKM2	Li, Lu, et al. (2022)
Nux Vomica	/	In silico	/	/	Regulated salivary secretion and cGMP‐PKG signaling pathway, modulated neuroactive ligand‐receptor interaction, serotonergic synapse and calcium signaling pathway	Zhang, Gai, et al. (2023)
*Eleutherococcus senticosus* (Rupr. & Maxim.) Maxim.	Leaves extract	In vivo	Two‐vessel occlusion (2‐VO) a total of two times in SD rats	Gavage: 100 mg/kg/day for 4 weeks after surgery	Modulated the disorder of bile acid metabolism, regulated the lipid disorder in the feces, alleviated the dysbiosis of gut microbiota by reducing *Proteobacteria*, *Enterobacter Escherichia‐Shigella* and *Oscillibacter*, and increasing the abundance *Faecalibacterium*, *Lactobacillus reuteri* , *Bacteroides* and *Clostridium butyricum*	Wang, Sun, et al. (2023)
Rhubarb	Water extract	In vivo	MCAO model in SD rats	Gavage: 100, 200 and 400 mg/kg/day at 0.5 h before surgery	Reduced the neurological deficit scores, rose the regional cerebral blood flow, decreased cerebral edema rate and infarct volume, decreased *Fournierella* and *Bilophila*, and increased *Enterorhabdus*, *Defluviitaleaceae*, *Christensenellaceae* and *Lachnospira*	Liu, Wang, et al. (2024)
Raw rhubarb	/	In vivo	MCAO/R model in SD rats	Gavage: 360, 540 and 810 mg/kg/day for 5 days before surgery	Attenuated cerebral infarct area, reduced inflammation, improved intestinal barrier function, attenuated gut microbiota dysbiosis, increased *Firmicutes*, and reduced *Proteobacteria*, *Escherichia*, *Shigella and Eubacterium coprostanoligenes* group	Xian et al. (2024)
*Cornus officinalis Sieb. et Zucc*. fruit	Cornuside	In vivo	MCAO/R model in SD rats	Gavage: 50, 100 and 200 mg/kg/day for 14 days after surgery	Reduced brain infarct volume, improved neurological function, reduced BBB and intestinal permeability, suppressed neuroinflammation and intestinal inflammation by improving the dysregulation of intestinal microflora, increasing the abundance of *Lachnospiraceae*, *Treponema*, *Lactobacillus*, *Ruminococcaceae* and *Prevotellaceae_NK3B31_group*, and decreasing the abundance of *Bacteroides*, and inhibiting the IL‐17F/TRAF6/NF‐κB pathway	Yan et al. (2024)
*Trillium tschonoskii* Maxim	Total saponins	In vivo	MCAO model in SD rats	Intragastrical administration: 100 mg/kg/day for 28 days after surgery	Alleviated cognitive dysfunction, suppressed apoptosis, and enhanced synaptic plasticity by activating the Shh signaling pathway	Wang, Tang, et al. (2023)
*Acorus tatarinowii*	Oils	In vivo	MCAO/R model in SD rats	Gavage: 5, 10 and 20 mg/kg/day for 7 days during reperfusion	Inhibited neuronal apoptosis, relieved cerebral infarction, degraded the release of IL‐6, TNF‐α, IFN‐γ and IL‐17, decreased *Verrucomicrobia*, *Akkermansia* and *Tenericutes*, increased *Tenericutes* and *Prevotella_copri*, and ameliorated the permeability of BBB	Huang et al. (2024)
*Ligusticum Chuanxiong* Hort.	/	In vitro and in silico	OGD model in SHSY‐5Y cells	Culture: 25, 50 and 100 μg/mL before OGD for 4 h	Decreased the protein expression of MMP9 and PTEN, and increased the protein expression of PPARα and TIMP1	Zhang, Cai, et al. (2023)
*Tricyrtis maculata* (D. Don) J. F. Macbr	Ferulic acid methyl ester	In vivo and in silico	MCAO model in SD rats	Administration: 12.5, 25 and 50 mg/kg/day for 7 days before surgery	Inhibited oxidative stress, promoted regeneration of vascular endothelial cells, and suppressed neuronal apoptosis via regulating the PI3K/HIF‐1α/VEGF pathway	Zhou, Yu, et al. (2024)
*Styphnolobium japonicum* (L.) Schott.	Sophoricoside	In vivo and in vitro	MCAO model in C57BL/6 mice; OGD/R model in primary neurons	Intraperitoneal injection: 45 and 90 mg/kg at 1 h before surgery; Culture: 25 and 50 μM for 30 min before OGD/R	Decreased edema and infarction volumes, reduced the neurological score, attenuated neuronal cell injury, suppressed apoptosis, and inhibited inflammation via activating the AMPK signaling pathway	Li, Zhang, et al. (2024)
*Phyllanthus urinaria*	/	In vivo	MCAO model in SD rats	Administration: 5 and 10 g/kg for 7 days before surgery	Inhibited inflammatory responses, improved neurological function, and reduced brain injury	Qin et al. (2024)
*Astragalus mongholicus* Bunge	Water extract	In vivo and in silico	MCAO/R model in SD rats	Intragastrical administration: 15, 30, 60 and 120 g/kg before surgery for 3 days, and after surgery at 1 h, 1 and 2 days	Inhibited apoptosis and oxidative stress, reduced Ca^2+^ overload and inflammation, and increased GSK3β, reduced PKCβ1, c‐Fos, Rela, NF‐κB p65 and IKKβ	Li, Lou, et al. (2024)
*Astragalus mongholicus* Bunge	Astragaloside IV	In vivo	MCAO model in SD rats	Intragastrical administration: 40 and 80 mg/kg/day at 6 h after ischemia for 15 days	Reduced infarct volume, enhanced nerve fibers reorganization, ameliorated brain microstructure damage, inhibited energy transporters, and reduced the expression of glycolytic rate‐limiting enzymes by activating AMPK	Li, Jia, et al. (2024)
*Achyranthes bidentata* Blume	Ecdysterone	In vivo and in vitro	MCAO model in SD rats; OGD/R model in PC12 cells	Administration: 20 and 40 mg/kg/day for 7 days before surgery; Culture: 20, 40 and 80 μM for 24 h after model	Reduced cerebral infarction volumes, decreased neurological impairment scores, inhibited oxidative stress, and reduced ferroptosis via ACSL4/NCOA4/FTH1 pathway	Sun et al. (2024)
*Ixeris sonchifolia (Bge.)* Hance	Luteolin‐7‐O‐β‐D‐glucuronide	In vivo and in vitro	tMCAO model in SD rats; OGD/R model in primary cerebral cortical	Tail vein injection: 0.24, 0.72 and 2.16 mg/kg after reperfusion; Culture: 0.37, 1.2, 3.7, 11, 33 and 100 μM for 36, 48 and 72 h during reperfusion	Improved the pathological injury degree, ameliorated cerebral edema, enhanced the cerebral nerve function score, reduced the permeability of the BBB, inhibited inflammatory response, decreased the infarct volume ratio by growing the expression of ZO‐1 and occludin, and reducing the expression of MMP9	Fan et al. (2024)
*Rhizoma Paridis*	Polyphyllin VII	In vivo and in vitro	MCAO model in C57BL/6 mice; Hippocampal neuron from MCAO mice; Murine CD4^+^ T cells	Intraperitoneal injection: 1 and 5 mg/kg/day for 14 days after surgery Culture: 0.5, 1 and 2 μM for 24 or 72 h	Decreased infarct volume, inhibited infiltration of Th1/Th17 cells, relieved brain injury, enhanced long‐term functional recovery, reduced neuroinflammation, improved Treg infiltration, inhibited ROS generation of T cells, and suppressed mTORC1 activation to influence glycolytic metabolism	Sang et al. (2024)
Probiotics						
Bifico	At least 1.0 × 10^7^ CFU *Entero‐coccus faecalis*, 1.0 × 10^7^ CFU *Lactobacillus acidophilus* , and 1.0 × 10^7^ CFU viable lyophilized *Bifidobacterium longum*	In vivo	pMCAO model in C57BL/6 mice	Gavage: 105 mg/kg/day, before surgery for 28 days, after surgery for 7, 14 and 28 days	Reduced cerebral infarction and promoted recovery of neurological function by regulating immunity and inflammation	Han et al. (2024)
*Limosilactobacillus* reuteri GMNL‐89 (G89) and *Lacticaseibacillus paracasei* GMNL‐133 (G133)	/	In vivo	pMCAO model in C57BL/6 mice	Gavage: G89, 8.2 × 10^7^ and 8.2 × 10^8^ CFU/kg; G133 8.2 × 10^7^ and 8.2 × 10^8^ CFU/kg; G89 + G133, 4.1 × 10^7^ and 4.1 × 10^7^ CFU/kg; G89 + G133, 4.1 × 10^8^ and 4.1 × 10^8^ CFU/kg; 4 day before surgery; at 3 h after surgery; for 6 days after surgery	Reduced the infarct volume, reduced the BBB permeability, conserved the tight junction integrity of gut barrier, attenuated inflammation, improved the gut dysbiosis, enhanced the neurological function, rose the quantities of *Lactonifactor*, *Coprococcus* and *Lachnospira*, and enriched the level of propionic acid	Wang, Liao, et al. (2025)
*Akkermansia muciniphila*	/	In vivo	Photothrombosis stroke model in C57BL/6 mice and CX3CR1^GFP/+^ mice	Gavage: 2 × 10^9^ CFU in 200 μL PBS once a day for 7 days before surgery	Decreased the infarct volumes and apoptotic cells, inhibited the activation of astrocytes and microglia, reduced the density of degenerating neurons, increased the expression of Arg1, CD206 and TGF‐β, and reduced the expression of IL‐6 and TNF‐α	Li, Ding, et al. (2024)
Prebiotics						
*Puerariae Lobatae* Radix‐resistant starch	/	In vivo	MCAO model in SD rats	Gavage: 125, 250 and 491.25 mg/kg/day for 14 days after surgery	Ameliorated gut barrier dysfunction, attenuated brain impairment, improved gut microbiota dysbiosis, and increased *Bifidobacterium*, *Akkermansia*, *Romboutsia* and *Butyricicoccus*	Lian et al. (2023)
Lactulose	Fructose and galactose	In vivo	Photothrombosis stroke model in C57BL/6 mice	Gavage: concentration of 15%, 150 μL per day for 14 days	Enhanced the functional outcome of stroke, rose anti‐inflammatory factors, amended gut microbiota dysbiosis, suppressed inflammatory reaction, improved metabolic disorder, and restored intestinal barrier injury	Yuan et al. (2021)

Abbreviations: 4‐HNE, 4‐hydroxynonenal; ACSL4, acyl‐coa synthetase long chain family member 4; AIF, allograft inflammatory factor 1; AKT, protein kinase B; AMPK, AMP‐activated protein kinase; AR, androgen receptor; Arg1, arginase 1; Bax, Bcl‐2 associated X protein; BBB, blood–brain barrier; BCCAO, bilateral carotid artery occlusion; Bcl‐2, B‐cell lymphoma‐2; BDNF, brain‐derived neurotrophic factor; Beclin‐1, myosin‐like Bcl‐2 interacting protein; caspase‐3, cysteinyl aspartate specific proteinase 3; caspase‐9, cysteinyl aspartate specific proteinase 9; CAT, catalase; CFU, colony‐forming units; cGMP, cyclic guanosine monophosphate; CRMP‐2, collapsin response mediator protein 2; EGCG (−)‐epigallocatechin‐3‐gallate; ERK, extracellular signal‐regulated protein kinase; FTH1, ferritin heavy chain 1; GLT‐1, glutamate transporter 1; GSH, glutathione; GSK‐3, glycogen synthase kinase‐3; GSK3β, glycogen synthase kinase 3 beta; HIF‐1α, hypoxia inducible factor 1 subunit alpha; HO‐1, heme oxygenase‐1; HSD11B1, hydroxysteroid 11‐beta dehydrogenase 1; IFN‐γ, interferon gamma; IGF1, insulin like growth factor 1; IKKβ, nuclear factor kappa B kinase subunit beta; IL‐17, interleukin‐17; IL‐17F, interleukin 17F; IL‐1β, interleukin‐1β; IL‐6, interleukin‐6; iNOS, inducible nitric oxide synthase; JAK2, janus kinase 2; Keap1, kelch‐like epichlorohydrin‐associated protein 1; LC3‐II, microtubule‐associated protein light chain 3 II; LDH, lactate dehydrogenase; MCAO/R, middle cerebral artery occlusion/reperfusion; MDA, malondialdehyde; MMP1, matrix metallopeptidase 1; MMP9, matrix metallopeptidase 9; MnSOD, manganese superoxide dismutase; MPO, myeloperoxidase; mTOR, mechanistic target of rapamycin kinase; NCOA4, nuclear receptor coactivator 4; NCXs, Na^+^/Ca^2+^ exchangers; NF‐κB, nuclear factor kappa‐B; NLRP3, NLR family pyrin domain containing 3; NO, nitric oxide; Nrf2, nuclear factor‐erythroid 2‐related factor‐2; OGD/R, oxygen glucose deprivation/re‐oxygenation; PARP1, poly (ADP‐ribose) polymerase 1; pdMCAO, permanently occlusion of distal middle cerebral artery; PI3K, phosphoinositide 3 kinase; PKCβ1, protein kinase C beta1; PKG, cGMP‐dependent protein kinase; PKM2, pyruvate kinase M2; PPARα, peroxisome proliferator activated receptor alpha; PPARγ, peroxisome proliferator‐activated receptor gamma; PTEN, phosphatase and tensin homolog; RIPK3, receptor interacting serine/threonine kinase 3; ROS, reactive oxygen species; Shh, sonic hedgehog; STAT3, signal transducer and activator of transcription 3; TBARS, thiobarbituric acid reactive substances; TIMP1, TIMP metallopeptidase inhibitor 1; TLR4, toll‐like receptor 4; TNF‐α, tumor necrosis factor α; TRAF6, TNF receptor associated factor 6; VCAM‐1, vascular cell adhesion molecule 1; VEGF, vascular endothelial growth factor; VEGFR2, vascular endothelial growth factor receptor 2; XO, xanthine oxidase; ZO‐1, zonula occludens‐1.

### 3.1 Inhibiting Oxidative Stress

Oxidative stress is the main pathological process connected with ischemic stroke. Therefore, it is very important to study antioxidant strategies for ischemic stroke (Pawluk et al. 2024). Many studies have shown that some dietary natural products have antioxidant activity that may alleviate ischemic stroke. For example, narirutin‐rich fraction from grape fruit peel was reported to reduce oxidative damage after cerebral IR injury in Wistar rats through elevating the activities of catalase (CAT) and superoxide dismutase (SOD), and decreasing malondialdehyde (MDA) level (Patel et al. 2022). Another study showed that *Tetrapleura tetraptera* fruit extract increased glutathione (GSH) level, CAT and SOD activities, reduced MDA level and xanthine oxidase (XO) activity in Wistar rats after cerebral IR injury (Saliu et al. 2021). Additionally, polyphenol‐rich fraction extract of *Thymus quinquecostatus* Celak. could reduce reactive oxygen species (ROS) level in SD rats with cerebral IR injury through activating the kelch‐like epichlorohydrin‐associated protein 1 (Keap1)/nuclear factor‐erythroid 2‐related factor‐2 (Nrf2)/heme oxygenase‐1 (HO‐1) signaling pathway (Fan et al. 2021).

In short, some dietary natural products and their components, such as *Tetrapleura tetraptera* fruit extract, polyphenol‐rich fraction extract of *Thymus quinquecostatus* Celak., and narirutin‐rich fraction from grape fruit peel, could enhance the activities of CAT, GSH and SOD, reduce the levels of MDA, XO and ROS, activate the Keap1/Nrf2/HO‐1 signaling pathway, thereby improving ischemic stroke.

### 3.2 Suppressing Inflammation

Inflammation is crucial in the pathogenesis of ischemic stroke and has a strong connection to secondary brain damage and adverse clinical outcomes. Thus, inhibiting inflammation has become a key target for therapeutic intervention on ischemic stroke (Cheng et al. 2022; Jayaraj et al. 2019). Some studies have shown that some dietary natural products could improve ischemic stroke by suppressing the inflammatory response. For instance, garcinol, isolated from fruit rind of *Garcinia indica* , could reduce cerebral IR injury‐induced tumor necrosis factor‐α (TNF‐α), interleukin (IL)‐1β and IL‐6 and through suppressing toll‐like receptor 4 (TLR4)/nuclear factor kappa‐B (NF‐κB) pathway in SD rats and PC12 cells (Kang et al. 2020). In addition, berberine from Rhizoma Coptidis exhibited neuroprotective effects during brain IR injury through suppressing NF‐κB‐mediated pyroptosis via growing peroxisome proliferator‐activated receptor gamma (PPARγ) (Zhao et al. 2021). Moreover, formononetin from red clover could decrease the levels of IL‐1β, IL‐6, TNF‐α and IL‐18 in SD rats with cerebral IR injury through restraining the janus kinase 2 (JAK2)/signal transducer and activator of transcription 3 (STAT3) signaling pathway (Yu et al. 2022). Besides, *Lithocarpus polystachyus* Rehd. ameliorated cerebral IR injury in SD rats via mitigating neuroinflammation via suppressing phosphoinositide 3 kinase (PI3K)/protein kinase B (AKT)/NF‐κB pathway and modulating pyroptosis mediated by NLR family pyrin domain containing 3 (NLRP3) (Liu, Wu, et al. 2024). Furthermore, puerarin cubic liquid crystal nanoparticles could enhance the bioavailability of puerarin in the blood, promote the distribution of puerarin in the brain, and against ischemic stroke in MACO rats by reducing the levels of IL‐1β, TNF‐α and IL‐6 (Chen et al. 2024). Moreover, curcumin‐loaded gelatin nanoparticles improved the solubility of curcumin and exhibited good dispersibility, and significantly reduced neuroinflammation in rats with cerebral IR injury (Yang et al. 2024).

In brief, some dietary natural products and their components, such as *Lithocarpus polystachyus* Rehd., garcinol from fruit rind of *Garcinia indica* , berberine from Rhizoma Coptidis and formononetin from red clover, could ameliorate ischemic stroke by reducing IL‐6, TNF‐α, IL‐18 and IL‐1β, inhibiting pyroptosis, and suppressing the JAK2/STAT3, TLR4/NF‐κB and PI3K/AKT/NF‐κB pathways.

### 3.3 Attenuating Excitotoxicity

Excitotoxicity, a specific type of glutamate‐mediated neurotoxicity, is associated with ischemia and neuronal damage. Mitigating excitotoxicity could prevent ischemic stroke‐induced damage (Lai et al. 2014). Several studies revealed that some dietary natural products could attenuate excitotoxicity to improve ischemic stroke. For instance, *Tetrapleura tetraptera* fruit extract could increase the activity of glutamine synthetase, thereby alleviating excitotoxicity induced by cerebral IR injury in Wistar rats via glutamate reuptake and/or glutamate clearance (Saliu et al. 2021). Additionally, emodin from *Rheum palmatum* L. increased the expression of glutamate transporter 1 (GLT‐1) and reduced the level of extracellular glutamate by activating the extracellular signal‐regulated protein kinase (ERK)‐1/2 pathway, thereby alleviating the neuronal damage after cerebral IR (Leung et al. 2020). Furthermore, olive oil and olive leaf extract alleviated excitotoxicity by increasing the expression of Na^+^/Ca^2+^ exchangers (NCXs), such as NCX1, NCX2 and NCX3 in Wistar rats, ultimately exerting a neuroprotective effect against cerebral ischemia (Khaksar et al. 2025).

In summary, some dietary natural products and their components, such as *Tetrapleura tetraptera* fruit extract, quercetin, emodin from *Rheum palmatum* L., olive oil and olive leaf extract, could mitigate excitotoxicity via enhancing glutamine synthetase activity, increasing the expression of GLT‐1, NCX1, NCX2 and NCX3, decreasing extracellular glutamate level, and activating the ERK‐1/2 pathway.

### 3.4 Regulating Gut Microbiota

Gut microbiota is associated with the risk of ischemic stroke and plays a vital role in the prognosis and recovery of ischemic stroke (Meng et al. 2023; Zhang, Jin, et al. 2023). Modulating the gut microbiota may become a new approach for ischemic stroke management (Zhao et al. 2023). Some dietary natural products have been reported to ameliorate ischemic stroke by targeting the gut microbiota. For instance, *Eleutherococcus senticosus* (Rupr. & Maxim.) Maxim. leaves could reduce post‐ischemic stroke injury in SD rats via the gut‐microbiota‐brain axis through reducing *Escherichia‐Shigella*, *Proteobacteria*, *Oscillibacter* and *Enterobacter*, while enriching *Faecalibacterium*, *Lactobacillus reuteri* , *Bacteroides* and *Clostridium butyricum* (Wang, Sun, et al. 2023). Another study revealed that water extract of rhubarb pretreatment could prevent ischemic stroke injury through modulating gut microbiota by decreasing *Fournierella* and *Bilophila*, and increasing *Enterorhabdus*, *Defluviitaleaceae*, *Christensenellaceae* and *Lachnospir* (Liu, Wang, et al. 2024). Moreover, *Acorus tatarinowii* oils could intervene in microglial phenotype and moderate neuronal damage in ischemic stroke SD rats via restoring gut microbiota composition, reducing *Verrucomicrobia*, *Akkermansia* and *Tenericutes*, and increasing *Tenericutes* and *Prevotella_copri* (Huang et al. 2024). Additionally, *Akkermansia muciniphila* administration reduced apoptotic cells and infarct volume, decreased the density of degenerating neurons, suppressed the activation of microglia, inhibited the activation of astrocytes, and increased multiple anti‐inflammatory factors after ischemic stroke (Li, Ding, et al. 2024). Furthermore, *Puerariae Lobatae* Radix‐resistant starch, a potential novel prebiotic, could alleviate ischemic stroke damage by rescuing gut microbiota dysbiosis and increasing the abundance of *Akkermansia* and *Bifidobacterium* (Lian et al. 2023).

In brief, some dietary natural products and their components, such as *Eleutherococcus senticosus* (Rupr. & Maxim.) Maxim. leaves, rhubarb water extract, *Acorus tatarinowii* oils, *Akkermansia muciniphila* and *Puerariae Lobatae* Radix‐resistant starch, showed a potent ability to modulate gut microbiota, and may help to alleviate ischemic stroke damage.

### 3.5 Protecting Blood–Brain Barrier

Blood–brain barrier (BBB) damage and dysfunction are important pathological features of ischemic stroke, which can lead to tissue damage, edema, inflammation and neural dysfunction (Lu and Wen 2024; Zheng et al. 2023). Therefore, protecting BBB has become an effective strategy for treating ischemic stroke. Some studies have revealed that some dietary natural products could protect BBB to alleviate ischemic stroke. For example, luteolin‐7‐O‐β‐d‐glucuronide from *Ixeris sonchifolia (Bge.)* Hance could alleviate cerebral IR injury by reducing the BBB permeability via increasing the protein expression of zonula occludens‐1 (ZO‐1) and occludin, while decreasing the expression of matrix metallopeptidase 9 (MMP9) (Fan et al. 2024). Additionally, cornuside from *Cornus officinalis Sieb. et Zucc*. fruit could restore BBB permeability induced by ischemic stroke in SD rats through increasing the protein expression of ZO‐1 and occludin (Yan et al. 2024). Meanwhile, *Acorus tatarinowii* oils could improve BBB permeability by growing the protein expression of ZO‐1 and occludin, thus alleviating ischemic stroke damage (Huang et al. 2024). Moreover, oridonin from Rabdosia rubescens exerted a protective effect after ischemic stroke by improving BBB integrity via upregulating the protein expression of ZO‐1, claudin‐5 and occludin (Li, Cheng, et al. 2021). Besides, withanolide A from *Withania somnifera* could reduce cerebral infarction, restore BBB disruption and ameliorate cerebral edema in Swiss albino mice after cerebral IR injury (Mukherjee et al. 2020).

In general, some dietary natural products and their components, such as *Acorus tatarinowii* oils, luteolin‐7‐O‐β‐d‐glucuronide from *Ixeris sonchifolia (Bge.)* Hance, withanolide A from *Withania somnifera* , cornuside from *Cornus officinalis Sieb. et Zucc*. fruit and oridonin from Rabdosia rubescens, have the potential to reduce BBB permeability and improve BBB integrity, and their potential mechanisms are related to increasing ZO‐1, claudin‐5 and occludin, and decreasing MMP9.

### 3.6 Promoting Angiogenesis

Angiogenesis is essential for restoring blood flow to damaged areas, promoting neural regeneration, and facilitating functional recovery. It is an important protective mechanism in the pathophysiology of ischemic stroke (Fang et al. 2023; Hu et al. 2024). Several studies showed that some dietary natural products could promote angiogenesis after ischemic stroke. For instance, astragaloside IV from *Astragalus mongholicus* Bunge enhanced angiogenesis in SD rats after cerebral IR injury via increasing the expression of brain‐derived neurotrophic factor (BDNF), insulin like growth factor 1 (IGF1) and vascular endothelial growth factor (VEGF), and activating PPARγ pathway (Li, Gan, et al. 2021). In addition, ferulic acid methyl ester from *Tricyrtis maculata* (D. Don) J. F. Macbr could alleviate cerebral IR injury in SD rats via promoting cerebral angiogenesis via regulating PI3K/hypoxia inducible factor 1 subunit alpha (HIF‐1α)/VEGF pathway (Zhou, Yu, et al. 2024). Moreover, salvianolic acid C from *Salvia miltiorrhiza* Bunge promoted angiogenesis by reducing the protein expression of vascular endothelial growth factor receptor 2 (VEGFR2), matrix metallopeptidase 1 (MMP1) and IGF1, and increasing the protein expression of PPARγ, ultimately alleviating ischemic stroke damage in SD rats (Yang, He, et al. 2021).

Overall, some dietary natural products and their components, such as astragaloside IV from *Astragalus mongholicus* Bunge, ferulic acid methyl ester from *Tricyrtis maculata* (D. Don) J. F. Macbr and salvianolic acid C from *Salvia miltiorrhiza* Bunge, could promote angiogenesis on ischemic stroke via enhancing the expression of BDNF, VEGF and PPARγ, decreasing the expression of VEGFR2 and MMP1, and regulating PI3K/HIF‐1α/VEGF pathway.

### 3.7 Inhibiting Autophagy

Autophagy plays a critical role in brain pathology after ischemic stroke. Therefore, regulating autophagy could serve as a promising approach for treating ischemic stroke (Wang, Fang, et al. 2021). Some dietary natural products have been reported to inhibit ischemic stroke by inhibiting autophagy. For example, oridonin from Rabdosia rubescens could inhibit autophagy by blocking the receptor interacting serine/threonine kinase 3 (RIPK3)/AMP‐activated protein kinase (AMPK)/Pink/Parkin signaling pathway, thereby rescuing early neuronal loss in ischemic stroke (Li, Song, et al. 2023). In another study, (−)‐epigallocatechin‐3‐gallate (EGCG) from green tea ameliorated cerebral IR injury in C57BL/6 mice and HT22 cells through inhibiting autophagy by regulating AKT/AMPK/mechanistic target of rapamycin kinase (mTOR) pathway (Wang et al. 2022). Additionally, cornin from *Verbena officinalis* L. could improve neurological recovery after cerebral IR injury in SD rats and U87 cells via suppressing autophagy through reducing the expression of microtubule‐associated protein light chain 3 II (LC3‐II) and myosin‐like Bcl‐2 interacting protein (Beclin‐1) by promoting the PI3K/AKT/mTOR pathway (Lan et al. 2022).

In a word, some dietary natural products and their components, such as oridonin from Rabdosia rubescens, EGCG from green tea and cornin from *Verbena offcinalis* L. could ameliorate ischemic stroke injury via inhibiting autophagy through reducing the expression of Beclin‐1 and LC3‐II, suppressing the RIPK3/AMPK/Pink/Parkin pathway, modulating the AKT/AMPK/mTOR pathway, and activating the PI3K/AKT/mTOR pathway.

### 3.8 Alleviating Apoptosis

Apoptosis is a key event in ischemic stroke injury, and inhibition of apoptosis could represent a promising therapeutic approach (Qin et al. 2022). A number of studies have shown that some dietary natural products could alleviate ischemic stroke by ameliorating apoptosis. For instance, indole‐3‐carbinol from cruciferous vegetables could inhibit neural cell apoptosis in SD rats after cerebral IR injury by increasing the expression of B‐cell lymphoma‐2 (Bcl‐2), while reducing the expression of Bcl‐2 associated X protein (Bax), cysteinyl aspartate specific proteinase (caspase)‐3 and caspase‐9 (Peng et al. 2024). In addition, cornin from *Verbena offcinalis* L. could alleviate cerebral IR injury through increasing the expression of Bcl‐2 and the apoptosis regulator ratio (Bcl‐2/Bax), while reducing the expression of Bax and cleaved caspase‐3 (Lan et al. 2022). Furthermore, sophoricoside from *Styphnolobium japonicum* (L.) Schott. inhibited neuronal apoptosis induced by cerebral IR injury through growing the expression of Bcl‐2, and reducing the expression of cleaved caspase‐3 and Bax via activating the AMPK pathway (Li, Zhang, et al. 2024).

In summary, some dietary natural products and their components, such as indole‐3‐carbinol from cruciferous vegetables, cornin from *Verbena offcinalis* L. and sophoricoside from *Styphnolobium japonicum* (L.) Schott., effectively prevented apoptosis after ischemic stroke through increasing the expression of Bcl‐2, enhancing the Bcl‐2/Bax ratio, reducing the expression of caspase‐9, Bax, cleaved caspase‐3 and caspase‐3, and activating the AMPK pathway.

### 3.9 Other Mechanisms

In addition to the aforementioned mechanisms, some dietary natural products may also improve ischemic stroke through other mechanisms. For example, apigenin from celery could prevent ischemic stroke by enhancing DNA repair via increasing the expression of DNA repair protein RAD51 homolog 1 (RAD51) and breast cancer type 1 susceptibility protein (BRCA1), while reducing DNA damage via reducing the expression of γH2A.X and P53‐binding protein 1 (53BP1) (Ping et al. 2024). In another study, ecdysterone from *Achyranthes bidentata* Blume improved ischemic stroke by suppressing ferroptosis via regulating acyl‐coa synthetase long chain family member 4 (ACSL4)/nuclear receptor coactivator 4 (NCOA4)/ferritin heavy chain 1 (FTH1) pathway (Sun et al. 2024). Additionally, total saponins from *Trillium tschonoskii* Maxim. Rhizome could promote post‐ischemic stroke functional recovery in SD rats through modulating oligodendrogenesis and axonal reorganization by regulating glycogen synthase kinase‐3 (GSK‐3)/β‐catenin/collapsin response mediator protein 2 (CRMP‐2) (Yang, Li, et al. 2021).

Some dietary natural products could alleviate ischemic stroke through inhibiting platelet aggregation and thrombosis (Kakarla et al. 2024). For instance, panax notoginseng triol saponins could reduce the area of cerebral infarction and water content of brain tissue in MCAO model rats by inhibiting platelet aggregation and thrombosis through regulating glycoprotein Ib‐α (Xu et al. 2021). Moreover, acteoside from *Osmanthus fragrans* (Thunb.) Lour. flowers attenuated cerebral IR injury in MCAO rats via inhibiting plasma kallikrein to regulate coagulation and suppress thrombus formation (Wang, Feng, et al. 2025). In addition, some dietary natural products attenuated ischemic stroke by improving mitochondrial function. A study found that ligustilide alleviated ischemic stroke injury in OGD/R model HT22 cells and MCAO model rats through promoting dynamin‐related protein 1‐mediated mitochondrial fission by activating the AMPK pathway (Wu, Liu, et al. 2022). Another study revealed that icariin from Epimedium attenuated cerebral IR injury in MCAO model rats via suppressing mitochondrial permeability transition pore opening (Zhou, Li, et al. 2024).

## 4 Clinical Trials

The Chinese Stroke Association recommends that primary prevention of stroke in high‐risk populations in China should focus on a diverse daily diet, increasing the intake of whole grains, fruits, vegetables, tubers, legumes, and dairy products, while limiting the intake of total fat and saturated fat (class I; evidence level A) (Wang et al. 2020). Several clinical trials have been carried out to investigate the effects of some dietary natural products on ischemic stroke (Table 3). For instance, a randomized, parallel, double‐blind, placebo‐controlled trial showed that 8‐week supplementation of curcumin‐piperine decreased the serum levels of triglycerides (TG), systolic and diastolic blood pressure, carotid intima‐media thickness (CIMT), total cholesterol (TC), weight, waist circumference, high‐sensitivity C‐reactive protein (hs‐CRP) in 66 patients with ischemic stroke, while increased total antioxidant capacity (TAC), and the levels of low‐density lipoprotein (LDL), serum fibrinogen, quality of life indicators and high‐density lipoprotein (HDL) showed no significant alterations (Boshagh et al. 2023). Additionally, pomegranate polyphenols could improve cognitive and functional recovery in 16 ischemic stroke inpatients based on a parallel, block‐randomized clinical trial (Bellone et al. 2019), which fell within the guideline (Wang et al. 2020). Moreover, a randomized clinical trial of 3 months intervention in 39 hospitalized patients with acute ischemic stroke found that treatment with aqueous extract of *Crocus sativus* L. reduced stroke severity during the first 4 days. The levels of serum S100 and neuron specific enolase (NSE) were reduced on the fourth day, while the concentration of BDNF was increased. The mean Barthel index was higher after 3 months (Asadollahi et al. 2019).

**TABLE 3:** Effects of dietary natural products on ischemic stroke from clinical studies.

Name	Study type	Subject	Dose & duration	Outcomes	Ref.
Curcumin‐piperine	Randomized, parallel, double‐blind, placebo‐controlled trial	66 patients with ischemic stroke	Oral administration: 505 mg/day (500 mg curcumin and 5 mg piperine) for 12 weeks	Reduced the serum levels of hs‐CRP (*p* = 0.026), TC (*p* = 0.009), TG (*p* = 0.001), CIMT (*p* = 0.002), weight (*p* = 0.001), waist circumference (*p* = 0.024), systolic and diastolic blood pressure (*p* < 0.001); increased TAC; no significant changes in serum fibrinogen, LDL, HDL and quality of life indicators	Boshagh et al. (2023)
Pomegranate polyphenols	Parallel, block‐randomized clinical trial	16 ischemic stroke inpatients	Oral administration: 1 g/time, twice per day for 7 days	Enhanced cognitive and functional recovery after ischemic stroke	Bellone et al. (2019)
Aqueous extract of *Crocus sativus* L.	Randomized clinical trial	39 acute ischemic stroke inpatients	Oral administration: 200 mg/day for 3 months	Reduced the levels of serum NSE and s100 on the fourth day; increased BDNF concentration on the fourth day; higher the mean Barthel index at the end of the 3‐month follow‐up	Asadollahi et al. (2019)

Abbreviations: CIMT, carotid intima‐media thickness; HDL, high‐density lipoprotein; hs‐CRP, high‐sensitivity C‐reactive protein; LDL, low‐density lipoprotein; NSE, neuron specific enolase; TAC, total antioxidant capacity; TC, total cholesterol; TG, triglycerides.

In general, some dietary products, such as curcumin‐piperine, pomegranate polyphenols and aqueous extract of *Crocus sativus* L., could improve ischemic stroke. In the future, more high‐quality clinical trials are need to be performed to assess the effects of more dietary natural products on ischemic stroke.

## 5 Conclusions and Future Perspectives

Ischemic stroke is ranked among the top causes of mortality and morbidity, and has become a major public health and socioeconomic burden globally. Epidemiological studies have shown that some dietary natural products, such as coarse grains, nuts and teas, could be potential modifiers associated with reduced ischemic stroke incidence. Experimental studies have revealed that many dietary natural products, such as fruits, vegetables, teas, herbs, probiotics and prebiotics, can alleviate ischemic stroke through suppressing oxidative stress, inhibiting inflammation, attenuating excitotoxicity, regulating gut microbiota, protecting BBB, promoting angiogenesis, inhibiting autophagy, alleviating apoptosis, suppressing platelet aggregation and thrombosis, and improving mitochondrial function. Several clinical trials have also suggested that some dietary natural products, such as curcumin‐piperine, pomegranate polyphenols and aqueous extract of *Crocus sativus* L., may have potential benefits in improving the prognosis of ischemic stroke.

In the future, the potential effect of more dietary natural products on ischemic stroke should be evaluated, with a focus on elucidating their mechanisms of action. In addition, the combined use of dietary natural products as well as their synergistic effects on ischemic stroke warrants further investigation. Furthermore, another promising avenue for research is the integration of dietary natural products with nanotechnology to enhance their bioavailability and facilitate their passage across the BBB, thereby improving the therapeutic potential of these substances for ischemic stroke. At the same time, more clinical trials need to be carried out to verify the effects and potential mechanisms of dietary natural products on ischemic stroke, with special attention to their potential side effects. Finally, effective dietary natural products could be developed into functional foods to prevent and manage ischemic stroke.

This paper is a comprehensive review and introduces the newest perspectives by integrating recent findings on dietary natural products against ischemic stroke, emphasizing emerging mechanisms. Moreover, it proposes future research direction. Furthermore, this review paper could be helpful for nutritionists, food researchers and the public to prevent and manage ischemic stroke by dietary natural products. Additionally, this review has several limitations. First, most of the available evidence is derived from the results of animal and cellular studies, while large‐scale, well‐designed clinical validation remains limited. Second, differences in study design, dosage, and preparation methods of dietary natural products limit the comparability and generalization of findings.

## Author Contributions

**Kai‐Yi Zhong:** conceptualization (lead), funding acquisition (supporting), investigation (lead), project administration (lead), writing – original draft (lead). **Yong Zhang:** investigation (equal), supervision (equal), visualization (equal), writing – review and editing (equal). **Nai‐Dong Wang:** investigation (equal), visualization (equal), writing – review and editing (equal). **Guang‐Wen Li:** investigation (equal), visualization (equal), writing – review and editing (equal). **Xian‐Jun Zhang:** investigation (equal), visualization (equal), writing – review and editing (equal). **Zhi‐Jun Yang:** conceptualization (equal), investigation (equal), project administration (equal), supervision (lead), writing – original draft (equal), writing – review and editing (lead).

## Funding

This study was supported by the Natural Science Foundation of Shandong Province of China (ZR2024QH427).

## Conflicts of Interest

The authors declare no conflicts of interest.

## Data Availability

Data sharing is not applicable to this article, as no datasets were generated or analyzed during the current study.
